# Marked Discordance Between Visual Field Loss and Optic Disc Cupping Revealing MOG Antibody-Associated Optic Neuritis in a Patient With Angle-Closure Glaucoma

**DOI:** 10.7759/cureus.114684

**Published:** 2026-08-17

**Authors:** Risa Mizumoto, Sotaro Mori, Makoto Nakamura

**Affiliations:** 1 Division of Ophthalmology, Department of Surgery, Kobe University Graduate School of Medicine, Kobe, JPN

**Keywords:** angle-closure glaucoma, differential diagnosis, magnetic resonance imaging, mog antibody-associated disease, optic neuritis, visual field

## Abstract

This study reports a case of MOG antibody-associated optic neuritis masquerading as acute angle-closure glaucoma in which marked discordance between optic disc cupping and visual field loss prompted magnetic resonance imaging (MRI) and led to the correct diagnosis.

A 69-year-old woman presented with acute ocular pain, decreased vision, and intraocular pressure exceeding 40 mmHg in the right eye. Humphrey visual field testing demonstrated nearly complete central visual field loss, whereas fundus examination revealed only mild optic disc cupping. Because the severity of visual field loss was disproportionate to the optic disc findings, concurrent optic neuritis was suspected. MRI demonstrated a hyperintense lesion in the right optic nerve on short tau inversion recovery (STIR) imaging. Intravenous methylprednisolone pulse therapy was initiated, followed by bilateral cataract surgery combined with ab interno microhook trabeculotomy because of persistent angle closure and anticipated prolonged corticosteroid therapy. Subsequent serological testing confirmed myelin oligodendrocyte glycoprotein antibody-associated disease (MOGAD). Five months later, visual acuity recovered to 20/20 with substantial improvement in the visual field, and intraocular pressure remained well controlled.

Even in patients with markedly elevated intraocular pressure suggestive of acute angle-closure glaucoma, pronounced discordance between optic disc findings and visual field loss should raise suspicion for concomitant optic neuritis or other nonglaucomatous optic neuropathies. MRI can facilitate the diagnosis of underlying MOG antibody-associated disease and guide appropriate neurological and ophthalmic management.

## Introduction

In the diagnosis of glaucoma, it is essential that findings from the optic nerve head examination, optical coherence tomography (OCT), and visual field testing are consistent with one another [1]. Although patients presenting with visual field defects are highly likely to have glaucoma because of its high prevalence, glaucoma is not the only cause of chronic, progressive visual field loss. Space-occupying lesions affecting the visual pathway should also be considered in the differential diagnosis [2].

Optic neuritis is an inflammatory disorder of the optic nerve that typically presents with acute or subacute visual loss, often accompanied by ocular pain, particularly with eye movement, impaired color vision, and a relative afferent pupillary defect. Myelin oligodendrocyte glycoprotein antibody-associated disease (MOGAD) is an inflammatory demyelinating disorder of the central nervous system in which optic neuritis is one of the most common clinical manifestations. Unlike glaucomatous optic neuropathy, in which structural optic nerve damage generally corresponds to characteristic visual field loss, acute inflammatory optic neuropathy may cause profound visual dysfunction despite relatively preserved optic disc morphology.

Conversely, conditions such as uveitic glaucoma [3] and angle-closure glaucoma [4] can cause acute visual impairment following an episode of markedly elevated intraocular pressure (IOP). In such cases, enlargement of the optic nerve head cupping may lag behind the severity of the visual field defect. Consequently, in patients with angle-closure glaucoma presenting with high intraocular pressure, a discrepancy between optic nerve head findings and visual field defects does not necessarily indicate an alternative diagnosis.

Herein, we report a patient with angle-closure glaucoma and markedly elevated intraocular pressure who simultaneously developed MOG antibody-associated optic neuritis. The coexistence of these conditions posed a diagnostic challenge because both can cause acute ocular pain and severe visual impairment. In this case, the marked discordance between profound visual field loss and relatively mild optic disc cupping, together with pain on eye movement, prompted magnetic resonance imaging (MRI) and subsequent neuroimmunological evaluation. This case highlights the importance of recognizing clinical findings that cannot be adequately explained by glaucoma alone, even when a clear glaucomatous mechanism for visual impairment is present.

## Case presentation

The patient was a 69-year-old woman who had been diagnosed with glaucoma 6 years earlier. Although IOP exceeding 30 mmHg had been documented in both eyes 5 years earlier and in the right eye 3 years earlier, it was successfully controlled to below 20 mmHg each time by adding antiglaucoma eye drops.

She subsequently developed a dull ache and decreased vision in her right eye and visited her previous ophthalmologist one week later. The IOP in the right eye was elevated to over 40 mmHg, and the central visual field was almost completely lost. A carbonic anhydrase inhibitor was added to her treatment regimen, and she was referred to our hospital for further evaluation and management.

**Figure 1:**
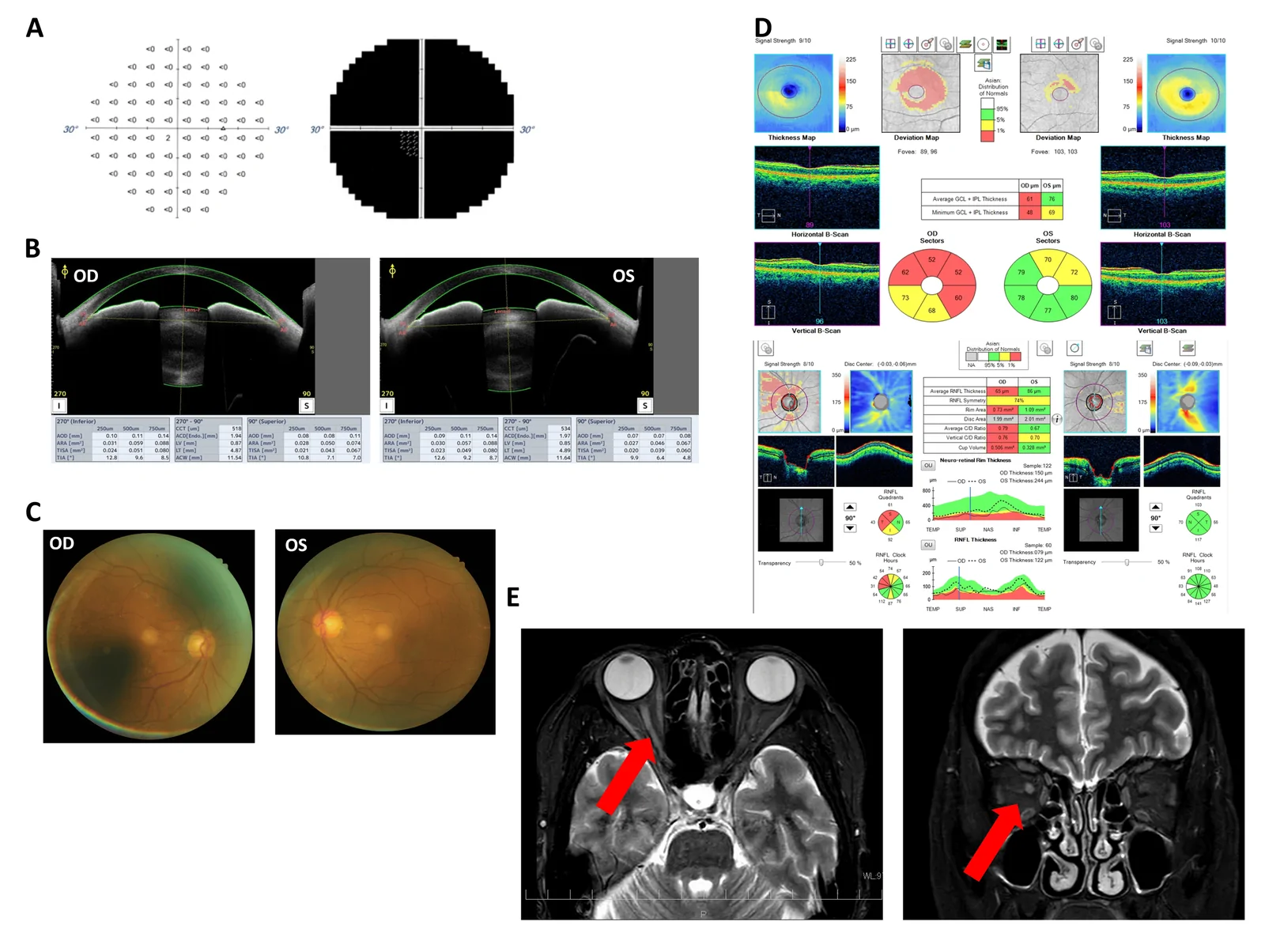
Ophthalmic and magnetic resonance imaging findings at presentation (A) Humphrey 30-2 visual field examination demonstrating profound visual field loss in the right eye. (B) Anterior segment optical coherence tomography demonstrating narrow anterior chamber angles in both eyes, supporting an angle-closure mechanism. (C) Color fundus photographs obtained without pharmacologic pupillary dilation because of concern for further IOP elevation in the presence of a markedly shallow anterior chamber. The apparent opacity over the macular/posterior pole region of the right eye represents an imaging artifact associated with the suboptimal image quality of nondilated fundus photography and does not represent a retinal lesion. (D) Macular ganglion cell-inner plexiform layer and circumpapillary retinal nerve fiber layer measurements obtained by optical coherence tomography. (E) Coronal and axial short tau inversion recovery magnetic resonance images demonstrating a hyperintense lesion in the anterior portion of the right optic nerve, supporting the diagnosis of optic neuritis. The arrow indicates the hyperintense lesion in the right optic nerve. OD, right eye; OS, left eye

At her initial visit to our hospital, best-corrected visual acuity (BCVA) was 20/2000 in the right eye and 20/16 in the left eye. A relative afferent pupillary defect was present in the right eye. IOP was 13 mmHg in the right eye and 16 mmHg in the left eye. Humphrey visual field testing (30-2), performed at the previous clinic, demonstrated unmeasurable sensitivity at almost all test points in the right eye, indicating profound visual field loss (Figure 1A). Gonioscopy revealed narrow angles, classified as Shaffer grade 1 and Scheie grade III, and the peripheral anterior chamber depth was approximately one-eighth of the corneal thickness according to the Van Herick method (Figure 1B). Axial length was 22.62 mm in the right eye and 22.92 mm in the left eye. Fundus examination revealed mild optic disc cupping in both eyes (Figure 1C). The average circumpapillary retinal nerve fiber layer thickness was 65 μm in the right eye and 86 μm in the left eye, while the average macular ganglion cell-inner plexiform layer thickness was 55 μm and 73 μm, respectively (Figure 1D). Although the narrow anterior chamber angles and history of markedly elevated IOP supported an angle-closure mechanism, several findings could not be adequately explained by glaucoma alone. These diagnostic red flags included profound visual field loss disproportionate to the relatively mild optic disc cupping, pain with eye movement, a relative afferent pupillary defect in the right eye, and a markedly reduced critical flicker frequency (CFF) of 5 Hz. These findings raised suspicion of a concurrent optic neuropathy, particularly optic neuritis, and MRI was therefore performed on the same day. Short tau inversion recovery (STIR) images demonstrated a hyperintense lesion in the anterior portion of the right optic nerve, suggestive of inflammation (Figure 1E). Accordingly, a diagnosis of right optic neuritis was made, and intravenous steroid pulse therapy was initiated the following day.

Following the initiation of steroid pulse therapy, the persistent ocular pain improved rapidly, beginning on the night of the first infusion. However, after acetazolamide was discontinued simultaneously, IOP increased to 29 mmHg in the right eye and 27 mmHg in the left eye on the second day of treatment, and further increased to 40 mmHg and 28 mmHg, respectively, on the third day, indicating bilateral IOP elevation. Although no moderate dilation of pupils was observed during daytime examinations, the patient reported recurrent ocular pain in the right eye during the night of the second day of steroid pulse therapy, raising concern for intermittent acute angle closure. Three days after initiation of steroid pulse therapy, bilateral cataract surgery combined with minimally invasive glaucoma surgery (*ab interno* microhook trabeculotomy) was performed. On the first postoperative day, IOP had decreased to 25 mmHg in the right eye and 14 mmHg in the left eye. For glaucoma management, only a fixed-combination eye drop containing a prostanoid receptor agonist and a β-blocker was resumed in both eyes. One treatment course consisted of intravenous methylprednisolone at 1,000 mg/day for three consecutive days, followed by oral prednisolone at 20 mg/day for three days. Visual acuity was reassessed at the completion of each course. Because visual acuity in the right eye remained impaired and had not recovered to logMAR 0, additional courses were administered while monitoring the clinical response. A total of three courses were ultimately completed, and the patient was discharged.

At the first postoperative follow-up visit, one month after surgery, BCVA had improved to 20/100 in the right eye and remained 20/13 in the left eye. IOP was 19 mmHg in the right eye and 18 mmHg in the left eye. Serum obtained before the initiation of intravenous methylprednisolone therapy was positive for myelin oligodendrocyte glycoprotein immunoglobulin G (MOG-IgG), measured using a live cell-based assay with an IgG1-specific secondary antibody. In this assay system, titers of ≥1:16 are reported as positive; however, the exact antibody titer in this patient was not available. Aquaporin-4 antibodies were negative by both cell-based assay and enzyme-linked immunosorbent assay. After acute treatment, oral prednisolone was continued at 20 mg/day and tapered by 5 mg per month according to our clinical treatment approach for optic neuritis associated with inflammatory demyelinating disease, as previously described [5]. Given the patient's pre-existing glaucoma and the known potential for corticosteroid-associated IOP elevation, IOP was carefully monitored during the tapering period.

**Figure 2:**
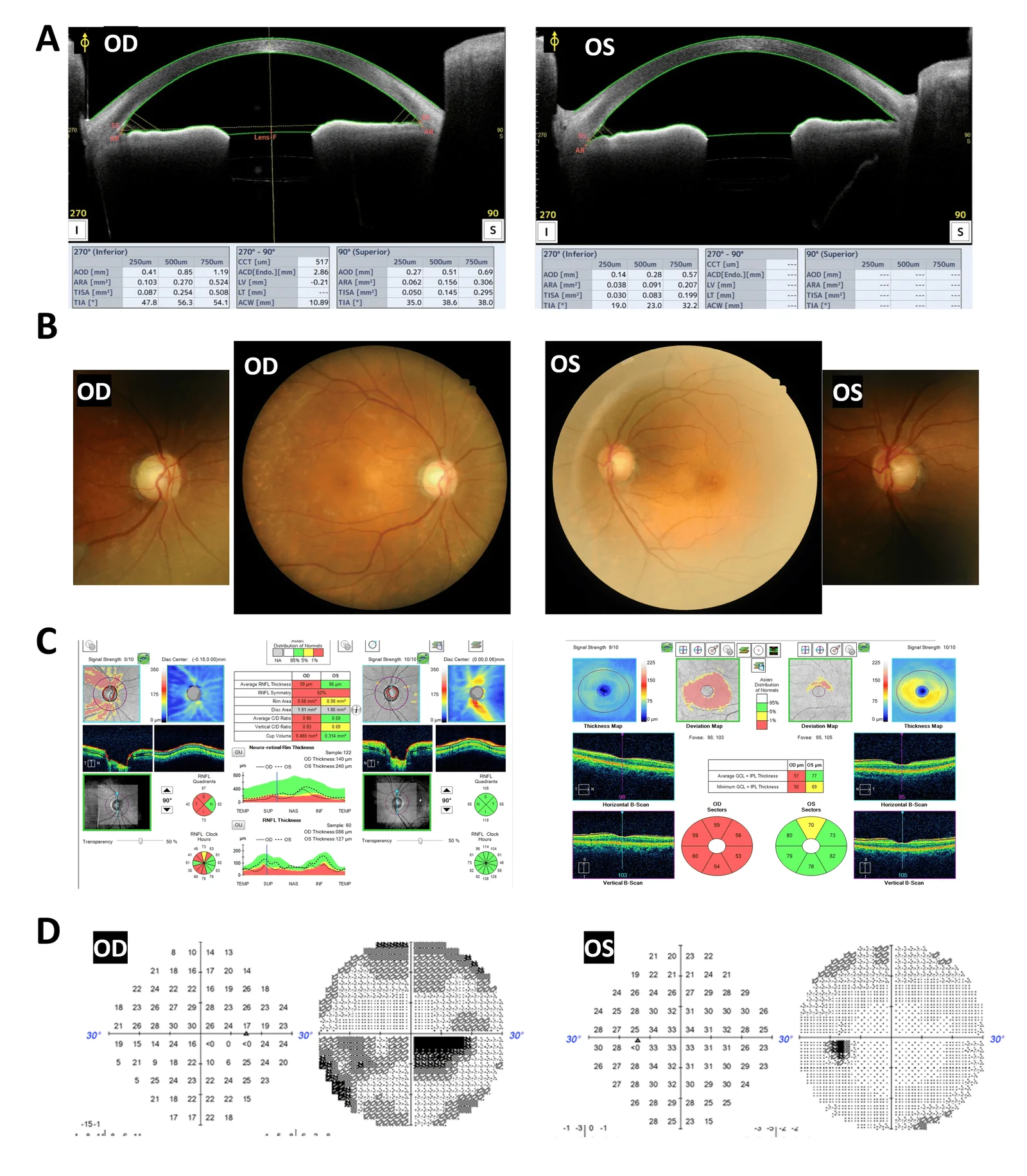
Ophthalmic findings five months after the initial presentation (A) Anterior segment optical coherence tomography demonstrating open anterior chamber angles in both eyes after cataract and glaucoma surgery. (B) Color fundus photographs demonstrating neuroretinal rim pallor in the right eye after optic neuritis. (C) Macular ganglion cell-inner plexiform layer and circumpapillary retinal nerve fiber layer measurements obtained by optical coherence tomography, demonstrating structural changes after the acute episode. (D) Humphrey 30-2 visual field examination demonstrating marked recovery of visual sensitivity in the right eye compared with the findings at presentation, although residual visual field defects remained. OD, right eye; OS, left eye

Five months after surgery, BCVA had further improved to 20/20 in the right eye and remained 20/13 in the left eye. IOP was well controlled at 14 mmHg in the right eye and 15 mmHg in the left eye, and the anterior chamber angles remained open (Figure 2A). Neuroretinal rim pallor was observed in the right eye and was considered a sequela of optic neuritis rather than glaucomatous optic neuropathy (Figures 2B, 2C). In contrast, the left eye showed optic disc cupping similar to that observed at the initial visit. Humphrey visual field testing demonstrated persistent residual visual field defects in the right eye, although marked improvement was observed compared with the pre-treatment findings (Figure 2D).

## Discussion

This case describes a patient who was initially referred for an acute IOP spike due to glaucoma but was ultimately found to have severe visual field loss caused by concurrent MOG antibody-positive optic neuritis. The principal clinical message from this case is that optic neuritis was suspected because of a marked discrepancy between the optic disc findings and the visual field defects. MRI supported the diagnosis of optic neuritis, and subsequent serological testing for MOG antibodies established the diagnosis of MOGAD in the appropriate clinical context.

The referring ophthalmologist had attributed the ocular pain to elevated IOP. However, the rapid resolution of pain following the initiation of steroid pulse therapy strongly suggests that the pain was primarily caused by optic neuritis. MOG antibody-positive optic neuritis has been reported to be associated with ocular pain more frequently than idiopathic optic neuritis or AQP4 antibody-positive optic neuritis [6], and the clinical presentation in the present case was consistent with this characteristic.

Furthermore, this case is significant because the episode of optic neuritis led to the diagnosis of MOGAD. Had the patient's visual impairment been attributed solely to angle-closure glaucoma, the underlying optic neuritis might have been overlooked, preventing appropriate immunological evaluation and treatment for MOGAD. As a result, the patient could have remained at risk for recurrent central nervous system inflammatory demyelinating events, including recurrent optic neuritis, myelitis, and encephalitis [7]. Because MOGAD can substantially affect long-term prognosis and quality of life, clinicians should consider the possibility of an underlying inflammatory demyelinating disease in patients with optic neuritis and perform an appropriate systemic evaluation, including antibody testing [5]. In the present case, the patient had no remarkable medical history other than appendicitis and no previous episodes suggestive of central nervous system demyelination.

The suspicion of optic neuritis in this case was prompted by the marked discrepancy between the optic disc findings and visual field defects. Optic disc cupping was only mildly enlarged despite unmeasurable sensitivity at almost all test points on the Humphrey 30-2 visual field examination. In general, the diagnosis of glaucoma is based on concordant findings of optic disc morphology, thinning of the retinal nerve fiber layer or macular inner retinal layers on optical coherence tomography (OCT), and characteristic visual field defects. However, in pediatric glaucoma, structural loss of retinal nerve fibers may be masked by enlargement of the scleral canal caused by elevated IOP, resulting in severe visual impairment despite relatively mild optic disc cupping [8]. Because deformation of the scleral canal secondary to elevated IOP can also occur in adults, structural damage and functional impairment do not necessarily progress in parallel. Moreover, previous studies have reported that no inter-eye difference in optic disc cupping is observed immediately after an acute angle-closure attack [9].

In the present case, however, the decisive factor leading to suspicion of optic neuritis was the marked discrepancy between the profound visual field loss-which could not be adequately explained by acute angle-closure glaucoma alone-and the relatively preserved optic disc appearance, despite approximately 10 days of markedly elevated IOP. Although similar discrepancies between optic disc findings and visual field defects have been reported in intracranial lesions masquerading as normal-tension glaucoma [10], reports of this pattern resulting from the coexistence of angle-closure glaucoma and acute optic neuritis are exceedingly rare.

The markedly reduced CFF of 5 Hz also supported the diagnosis. Although reduced CFF has been reported in glaucoma, values generally remain in the range of 30-40 Hz [11]. Therefore, the profound reduction observed in the present case is difficult to explain by glaucoma alone and strongly suggests the presence of an additional optic neuropathy. Accordingly, even in patients presenting with markedly elevated IOP, clinicians should consider measuring CFF and performing MRI of the optic nerve when visual field loss is disproportionately severe relative to the optic disc findings. 

Another clinical challenge in this case was determining the optimal surgical strategy after bilateral IOP elevation developed during steroid pulse therapy. Although angle closure was considered the principal mechanism underlying the marked IOP elevation, a steroid-induced component could not be completely excluded, and prolonged systemic corticosteroid treatment was anticipated. Therefore, cataract surgery was performed to eliminate the angle-closure mechanism, together with ab interno microhook trabeculotomy to facilitate aqueous outflow and reduce the risk of subsequent IOP elevation during corticosteroid therapy. Following surgery, IOP was carefully monitored throughout systemic corticosteroid treatment and remained well-controlled, including during continued oral prednisolone therapy. At that time, the patient--an elderly woman with optic neuritis and a BCVA of 20/2000 in the affected eye--was also suspected of having neuromyelitis optica spectrum disorder (NMOSD), and AQP4 antibody testing had been performed, although the results were not yet available. Because patients with NMOSD generally require prolonged corticosteroid therapy [12], we performed cataract surgery to eliminate the angle-closure mechanism and simultaneously performed ab interno microhook trabeculotomy to reduce the risk of future steroid-induced IOP elevation. Simultaneous bilateral glaucoma surgery is generally avoided because postoperative complications may result in bilateral visual impairment [13]. However, in the present case, the right eye had already lost central visual function, whereas the left eye was the patient's only functionally useful eye. Given the bilateral IOP elevation, rapid IOP control was considered the highest priority, and simultaneous bilateral surgery was therefore performed. Fortunately, postoperative hyphema was minimal, and postoperative visual function was preserved.

## Conclusions

We report a rare case of angle-closure glaucoma with an acute IOP spike complicated by MOG antibody-associated optic neuritis. Even when markedly elevated IOP and an angle-closure mechanism are present, visual dysfunction that is disproportionate to optic disc findings should prompt consideration of a concurrent nonglaucomatous optic neuropathy. In such clinically discordant cases, neuro-ophthalmic assessment, including critical flicker frequency testing and MRI of the optic nerve, may be considered. Because multiple medical and surgical interventions were performed within a short period in this single case, the respective contributions of optic neuritis, angle closure, IOP reduction, cataract surgery, and corticosteroid treatment to the visual course cannot be fully separated.
